# Quality of life among adolescents with sickle cell disease: Mediation of pain by internalizing symptoms and parenting stress

**DOI:** 10.1186/1477-7525-6-60

**Published:** 2008-08-09

**Authors:** Lamia P Barakat, Chavis A Patterson, Lauren C Daniel, Carlton Dampier

**Affiliations:** 1 Department of Psychology, Drexel University, Philadelphia, Pennsylvania, USA; 2 Marian Anderson Comprehensive Sickle Cell Center of St. Christopher's Hospital for Children and Department of Pediatrics, Drexel University College of Medicine, Philadelphia, Pennsylvania, USA

## Abstract

**Background:**

This study aimed to clarify associations between pain, psychological adjustment, and family functioning with health-related quality of life (HRQOL) in a sample of adolescents with sickle cell disease (SCD) utilizing teen- and parent-report.

**Methods:**

Forty-two adolescents (between the ages of 12 and 18) with SCD and their primary caregivers completed paper-and-pencil measures of pain, teen's psychological adjustment, and HRQOL. In addition, primary caregivers completed a measure of disease-related parenting stress. Medical file review established disease severity.

**Results:**

Pearson correlations identified significant inverse associations of pain frequency with physical and psychosocial domains of HRQOL as rated by the teen and primary caregiver. Generally, internalizing symptoms (i.e. anxiety and depression) and disease-related parenting stress were also significantly correlated with lower HRQOL. Examination of possible mediator models via a series of regression analyses confirmed that disease-related parenting stress served as a mediator between pain frequency and physical and psychosocial HRQOL. Less consistent were findings for mediation models involving internalizing symptoms. For these, parent-rated teen depression and teen anxiety served as mediators of the association of pain frequency and HRQOL.

**Conclusion:**

Results are consistent with extant literature that suggests the association of pain and HRQOL and identify concomitant pain variables of internalizing symptoms and family variables as mediators. Efforts to improve HRQOL should aim to address internalizing symptoms associated with pain as well as parenting stress in the context of SCD management.

## Background

Although resilient psychological functioning in pediatric sickle cell disease (SCD) is now considered the norm [1], there is also general agreement that health-related quality of life (HRQOL) is impaired among these youth [2]. Limitations in HRQOL have been documented consistently for youth with SCD [3,4], particularly as children move into adolescence and young adulthood [5,6]. Sickle cell pain, a common manifestation that is recurrent, acute, and unpredictable, may be the most important disease complication associated with decrements in physical and psychosocial domains of HRQOL [3,7,8]. However, the connection of pain with functioning across domains of HRQOL in SCD is not firmly established.

In the biobehavioral model of pain, a number of variables, in addition to disease severity, influence pain perception [9]. These variables, such as functional status, pain coping, family environment, social support, and psychological adjustment, are potentially modifiable. Pain antecedents, pain concomitants (such as depression and anxiety), and pain consequences (such as psychosocial functioning and disability) are also identified within the model. Based on the biobehavioral model of pain and conclusions of a review of quality of life assessment for children [10], we posit that concomitant variables such as family environment (including parenting stress associated with disease-related events) and psychological functioning (namely internalizing symptoms) may mediate the association of pain with teen- and parent-reports of HRQOL (considered a pain consequence) in pediatric SCD.

A number of disease-related factors have been found to affect HRQOL in pediatric SCD. Fuggle and colleagues [3] found that sickle cell pain was associated with decrements in social and recreational functioning as well as school attendance as ascertained through pain diaries completed over a one month period in a sample of 25 children with SCD. The results of other studies confirm that higher pain levels are associated with decrements in participation in activities [8,11] and in school attendance [12]. To extend the association of pain with HRQOL, Palermo and colleagues [5] documented sickle cell complications (including pain), as well as child age and gender, as central to physical but not psychosocial HRQOL in their sample of youth with SCD. Yet, Panepinto and colleagues found that only pain, not other SCD complications, was associated with the physical domain of HRQOL but not the psychosocial domain [4]. Others identify decrements in social and school competence for children with SCD, compared to peers, but do not find an association with disease severity measured as sickle cell type [13].

Although pain and other sickle cell complications show an association with decrements in engagement in physical activities and in physical domains of HRQOL, documentation of a significant association of pain with psychosocial domains of HRQOL is not consistent [14]. Researchers suggest that a number of variables may influence pain perception and HRQOL, such as socioeconomic status [14], internalizing symptoms among youth [14-16], and disease-related parenting stress [17,18]. Additionally, using daily pain diaries, Gil and colleagues demonstrated that pain predicted decrements in positive mood and higher levels of stress. An association of negative mood and stress with same day pain ratings was also identified [19]. However, there are no clear findings regarding the role of concomitant pain variables as mediators of the association of pain and HRQOL in pediatric SCD [16].

Given limitations in HRQOL experienced by youth with SCD [3,4], the importance of HRQOL in assessment of outcomes of medical treatments [10], and the critical juncture of adolescence in terms of successful transition [20], we aimed to examine the role of psychological adjustment (i.e. internalizing symptoms of anxiety and depression) and family functioning (i.e. disease-related parenting stress) in the association of pain with HRQOL. If these concomitant pain variables are indeed central to understanding HRQOL, by targeting internalizing symptoms and/or family functioning, we may better support adolescents with SCD in managing their condition as they transition to adult responsibilities and healthcare services [20]. Adolescents with SCD and their caregivers completed measures to obtain a broad description of HRQOL among these youth and account for documented variations in HRQOL by reporter [4,10,21-23]. We expected that pain frequency would be associated with lower scores on physical and psychosocial domains of HRQOL. Moreover, we hypothesized that internalizing symptoms of the adolescent with SCD and disease-related parenting stress would mediate the association of pain with HRQOL.

## Methods

### Participant Recruitment

The current data were collected as part of a larger study examining risk and resistance in health outcomes for adolescents with sickle cell disease (SCD). Detailed descriptions of the participant recruitment and procedures are given in previous publications [17]. All patients at an urban Comprehensive Sickle Cell Center between the ages of 12–18 were eligible for the study with the exclusion if English was not their first language. Eligible patients were contacted by clinic staff during clinic visits or by telephone about participation. Of 71 eligible participants who were contacted regarding the study, 44 agreed to participate (participation rate = 61%). The sample for this study comprised 42 adolescent-primary caregiver pairs, for whom complete data were available.

Demographic and disease-related information regarding the sample may be found in Table 1. Information is not available on non-participants, who noted lack of interest as the primary reason for lack of participation in the study. However, characteristics of the adolescent sample are representative of the Comprehensive Sickle Cell Center from which they were drawn in that approximately half of the patient participants were female (50%), most had SCD-SS (66.7%), most identified as African-American (88.1%). Most primary caregivers had a high school education or some college (71.4%), and family income was primarily under $20,000/year (28.9%) or between $20,000 and $50,000/year (52.6%).

**Table 1:** Sample demographics.

	*N*^a ^(%)	*M *(*SD*)	*Range*
Age Teen		15.00 (1.82)	12.00–19.00
Primary Caregiver		44.12 (10.19)	
Gender (Female) Teen	21 (50.00)		
Primary Caregiver	34 (81.00)		
Grade		8.71 (1.85)	5^th^-1^st ^year college
Ethnicity Teen			
African American	37 (88.10)		
Other	5 (11.90)		
Ethnicity Primary Caregiver			
African American	35 (83.30)		
Other	6 (14.30)		
Primary Caregiver Education			
1^st^–8^th ^grade	2 (4.80)		
9^th^–12^th ^grade	14 (33.30)		
Some college/Vocational	16 (38.10)		
College	5 (11.90)		
Professional/Graduate	5 (11.90)		
Income			
< $19,999	11 (28.90)		
$20,000–49,999	20 (52.60)		
$50,000+	7 (18.40)		
SCD Type (SCD-SS)	28 (66.70)		

^a^*N *= 42

### Measures

#### Varni Pediatric Pain Questionnaire [24]

(PPQ) is a patient- and parent-report pain rating scale for current pain and worst pain ever felt using questions in varying response formats. Pain frequency (*α*_*pc *_= .94; *α*_*teen *_= .87), noted by 7-point Likert-type responses to three questions, was used to reflect pain.

#### The Behavioral Assessment System for Children [25]

(BASC) is a self-, teacher-, and parent-report measure of adaptive and clinical functioning for children and adolescents ages 2 1/2 to 18 years of age. The questions address emotions, behaviors, and self-perceptions and produce composite scores. Scores are converted into t-scores, with scores less than 67 deemed in the normal range, 67–70 as borderline clinical, and above 70 as within the clinical range [25]. For this study, anxiety and depression subscales from the primary caregiver (*α*_*Anx *_= .83; *α*_*Dep *_= .82) and teen (*α*_*Anx *_= .79; *α*_*Dep *_= .86) versions were used.

#### Pediatric Inventory for Parents [26]

(PIP) is a 42-item measure completed by the primary caregiver regarding stress associated with caring for a child with a chronic illness. Caregivers respond to questions on a 5-point Likert-type scale about frequency and difficulty of events in the domains of communication, emotional functioning, medical care, and role function. The total difficulty score (PIP-D; α = .96), which reflects strain in the parent-child relationship due to disease-related events, was used in the analyses.

#### Child Health Questionnaire-50

(CHQ) is a 50-item measure used to assess physical, health, and social well being of children ages 5–18 using parent- and child-report [27]. Primary caregivers and teens responded to each question based on 4 to 6 continuous anchor responses. This measure has been validated in a group of children and adolescents with SCD [28,29], and has age appropriate norms for the subscales and summary scores. From primary caregiver and adolescent report versions, the physical functioning scale reflected physical domain of HRQOL and the self-esteem scale reflected psychosocial domain.

Demographic information was gathered based on primary caregiver response to a *General Information Form. Medical File Review *was used to assess occurrence of pain episodes and acute chest syndrome (among the most frequent and severe complications of SCD) to determine disease severity using a weighted score as outlined by Day [30]. The *Risk Index *[31] is well-established and assesses sociodemographic and psychological risk based on social and familial risk factors. The scored measure, for this study, yielded a risk index based on presence of six specific risk factors (i.e. single-parent household, maternal caregiver with less than high school education, ethnic minority status, large family size, family conflict, and maternal psychological distress).

### Procedure

Families were given the option to complete the study measures in their home or at the Sickle Cell Center. Caregivers and teens provided informed consent/assent before beginning the paper and pencil measures, which took approximately 2–3 hours to complete. Participants were given the option of having research assistants read questionnaires aloud. Data were collected from July 2003 through March 2006 by the study investigators and trained research assistants (teams of doctoral students and advanced undergraduates). The protocol was approved by the appropriate Institutional Review Board.

### Data Analysis

Preliminary analyses involved assessing the associations of demographic variables (child age, primary caregiver education, family income, risk index, and disease severity) with parent- and teen-reported PPQ pain frequency, primary caregiver- and teen-reported BASC anxiety and BASC depression, PIP difficulty, and primary caregiver- and teen-reported health-related quality of life (HRQOL) based on CHQ physical functioning and CHQ self-esteem to determine the need for covariates. In addition, preliminary Pearson correlations were computed among the variables under study to assess criteria for mediation and potential mediator models. Mediation criteria include: (1) pain is correlated with the mediator (internalizing symptoms, disease-related parenting stress); (2) pain is correlated with HRQOL; (3) the mediator is correlated with HRQOL; and (4) the mediator accounts for the association of pain with HRQOL (i.e. the association of pain with HRQOL is reduced when the mediator is included in the model). Subsequently, where appropriate, regression models were computed to test mediation based on a procedure described by Baron and Kenny [32] as were follow-up Sobel's tests of the indirect effect.

## Results

### Variable Description

Descriptive information for the variables under study is provided in Table 2. Primary caregivers and teens reported infrequent pain and mild to moderate pain intensity. Caregiver and teen report of BASC anxiety, BASC depression and physical functioning and self-esteem health-related quality of life (HRQOL) scores were within the normative range. T-tests were conducted to compare parent and teen report on all measures with parallel forms. Only one significant difference emerged on reports of physical functioning on the CHQ as teens reported significantly higher physical functioning [*t*(80) = -2.68, *p *= .009]. PIP difficulty was significantly lower than levels reported in other pediatric samples [*t*(39) = -2.77, p = .009] [17].

**Table 2:** Description of variables under study

***Variables***	***Mean***	***SD***	***Range***
*Parent*			
PPQ Pain Frequency	2.56	2.07	0.00–7.00
BASC Anxiety	50.05	11.18	33.00–79.00
BASC Depression	48.51	9.92	35.00–75.00
PIP-Difficulty	97.87	33.19	47.00–175.00
CHQ Physical Functioning	56.91	32.51	0.00–100.00
CHQ Self Esteem	70.04	23.86	16.67–100.00
			
*Child*			
PPQ Pain Frequency	2.41	1.99	0.00–7.00
BASC Anxiety	49.98	8.67	34.00–70.00
BASC Depression	50.71	9.57	43.00–74.00
CHQ Physical Functioning	73.98	24.69	14.81–100.00
CHQ Self Esteem	73.61	17.54	33.93–100.00

PPQ = Pediatric Pain Questionnaire; BASC = Behavioral Assessment System for Children; PIP = Pediatric Inventory for Parents; CHQ = Child Health Questionnaire

### Preliminary Analyses

Pearson correlations of demographic variables with the variables under study showed only two significant correlations. Risk index was associated with teen-reported PPQ pain frequency (*r *= .33, *p *= .034) and disease severity was associated with parent-reported BASC anxiety (*r *= .34, *p *= .035); therefore, risk index and disease severity were controlled in appropriate analyses. For sickle cell type, an ANOVA was conducted to examine differences on variables under study among teens; there were no significant differences.

### Mediation Analyses

Preliminary Pearson correlations (see Table 3) supported a number of mediation models including PIP difficulty as the mediator for: (1) Primary caregiver PPQ pain frequency → caregiver CHQ physical functioning/caregiver CHQ self-esteem/teen CHQ physical functioning; (2) Teen PPQ pain frequency → caregiver CHQ physical functioning/teen CHQ physical functioning/teen CHQ self-esteem.

**Table 3:** Preliminary mediation correlations

	**2**	**3**	**4**	**5**	**6**	**7**	**8**	**9**	**10**	**11**
1. PPQ PC Pain Frequency	0.58**	0.21	0.28^†^	-0.07	-0.13	0.48**	-0.48**	-0.39*	-0.52**	-0.12
2. PPQ Teen Pain Frequency		0.17	0.30^†^	0.35*	0.14	0.43**	-0.63**	-0.24	-0.71**	-0.37*
3. BASC PC Anxiety			0.72**	0.30^†^	-0.06	0.40*	-0.37*	-0.40*	-0.39*	-0.21
4. BASC PC Depression				0.28^†^	0.17	0.33*	-0.32*	-0.42**	-0.59**	-0.30^†^
5. BASC Teen Anxiety					0.27^†^	0.21	-0.25	-0.03	-0.46**	-0.33*
6. BASC Teen Depression						-0.07	0.09	-0.21	0.01	-0.43*
7. PIP Difficulty							-0.52**	-0.49**	-0.57**	-0.21
8. HQ PC Physical Functioning								0.28^†^	0.57**	0.10
9. CHQ PC Self Esteem									0.17	0.46**
10. CHQ Teen Physical Functioning										0.22
11. CHQ Teen Self Esteem										

† *p *= .10, * *p *< .05, ** *p *< .01

PC = Primary Caregiver; PPQ = Pediatric Pain Questionnaire; BASC = Behavioral Assessment System for Children; PIP = Pediatric Inventory for Parents; CHQ = Child Health Questionnaire.

For internalizing symptoms, mediation models receiving preliminary support for primary caregiver-reported BASC depression: (1) Primary caregiver PPQ pain frequency → caregiver CHQ physical functioning/teen CHQ physical functioning/caregiver CHQ self-esteem/teen CHQ self-esteem; (2) Teen PPQ pain frequency → caregiver CHQ physical functioning/caregiver CHQ self-esteem/teen CHQ physical functioning/teen CHQ self-esteem. Also, there was preliminary support for teen-reported BASC anxiety: Teen PPQ pain frequency → caregiver CHQ physical functioning/teen CHQ physical functioning/teen CHQ self-esteem. No mediation models involving caregiver-reported BASC anxiety or teen-reported BASC depression were supported in these preliminary analyses. Tables 4 and 5 present results of the mediation regression analyses and the follow-up tests.

**Table 4:** Mediation analyses for PIP difficulty as mediator

**Model**	**Predictor**	**Outcome Variable**	***β***	***p***	**Criterion** ** Met**
	**PC PPQ Frequency**	**PIP Difficulty**	**.48**	**.002**	

1	PC PPQ Frequency	CHQ PC Physical Functioning	-.48	.001	z = -2.01 *p *= .022
	PC PPQ Frequency PIP Difficulty	CHQ PC Physical Functioning	-.40	.017	
2	PC PPQ Frequency	CHQ PC Self-esteem	-.39	.011	z = -1.94 *p *= .026
	PC PPQ Frequency PIP Difficulty	CHQ PC Self-esteem	-.38	.022	
3	PC PPQ Frequency	CHQ Teen Physical Functioning	-.52	< .001	z = -2.18 *p *= .014
	PC PPQ Frequency PIP Difficulty	CHQ Teen Physical Functioning	-.44	.006	

	**Teen PPQ Frequency Risk Index**	**PIP Difficulty**	**.37**	**.026**	

4	Teen PPQ Frequency Risk Index	CHQ PC Physical Functioning	-.63	< .001	z = -1.57 *p *= .057
	Teen PPQ Frequency PIP Difficulty Risk Index	CHQ PC Physical Functioning	-.31	.040	
5	Teen PPQ Frequency Risk Index	CHQ Teen Physical Functioning	-.75	< .001	z = -1.80 *p *= .036
	Teen PPQ Frequency PIP Difficulty Risk Index	CHQ Teen Physical Functioning	-2.83	.008	
6	Teen PPQ Frequency Risk Index	CHQ Teen Self-esteem	-.30	.063	No
	Teen PPQ Frequency PIP Difficulty Risk Index	CHQ Teen Self-esteem	-.06	-.344	

PC = Primary Caregiver; PPQ = Pediatric Pain Questionnaire; BASC = Behavioral Assessment System for Children; PIP = Pediatric Inventory for Parents; CHQ = Child Health Questionnaire.

**Table 5:** Mediation analyses for BASC internalizing problems as mediator

**Model**	**Predictor**	**Outcome Variable**	***β***	***p***	**Criterion** ** Met**
	**PC PPQ Frequency**	**BASC PC Depression**	**.28**	**.082**	

7	PC PPQ Frequency PC PPQ Frequency BASC PC Depression	CHQ PC Physical Functioning CHQ PC Physical Functioning	-.48 -.22	.001 .175	No
8	PC PPQ Frequency	CHQ PC Self-Esteem	-.40	.011	z = -1.41 *p *= .080
	PC PPQ Frequency BASC PC Depression	CHQ PC Self-Esteem	-.34	.029	
9	PC PPQ Frequency	CHQ Teen Physical Functioning	-.52	< .001	z = -1.62 *p *= .052
	PC PPQ Frequency BASC PC Depression	CHQ Teen Physical Functioning	-.50	.001	
10	PC PPQ Frequency	CHQ Teen Self-Esteem	-.12	.467	z = -1.23 *p *= .109
	PC PPQ Frequency BASC PC Depression	CHQ Teen Self-Esteem	-.29	.099	

	**Teen PPQ Frequency**^a^	**BASC PC Depression**	.27	.116	

11	Teen PPQ Frequency	CHQ PC Physical Functioning	-.63	< .001	No
	Teen PPQ Frequency BASC PC Depression	CHQ PC Physical Functioning	-.17	.257	
12	Teen PPQ Frequency	CHQ PC Self-Esteem	-.17	.298	z = -1.32 *p *= .093
	Teen PPQ Frequency BASC PC Depression	CHQ PC Self-Esteem	-.39	.019	
13	Teen PPQ Frequency	CHQ Teen Physical Functioning	-.75	< .001	z = -1.45 *p *= .076
	Teen PPQ Frequency BASC PC Depression	CHQ Teen Physical Functioning	-.43	< .001	
14	Teen PPQ Frequency	CHQ Teen Self-Esteem	-.30	.063	No
	Teen PPQ Frequency BASC PC Depression	CHQ Teen Self-Esteem	-.17	.302	

	**Teen PPQ Pain Frequency**^a^	**BASC Teen Anxiety**	.30	.067	

15	Teen PPQ Frequency	CHQ Teen Physical Functioning	-.75	< .001	z = -1.36 *p *= .086
	Teen PPQ Frequency BASC Teen Anxiety	CHQ Teen Physical Functioning	-.24	.058	
16	Teen PPQ Frequency	CHQ Teen Self-esteem	-.30	.063	No
	Teen PPQ Frequency BASC Teen Anxiety	CHQ Teen Self-esteem	-.20	.218	

^a ^Risk Index controlled in analyses. PC = Primary Caregiver; PPQ = Pediatric Pain Questionnaire; BASC = Behavioral Assessment System for Children; PIP = Pediatric Inventory for Parents; CHQ = Child Health Questionnaire.

#### PIP difficulty as mediator

PIP difficulty served as a mediator between primary caregiver PPQ pain frequency with primary caregiver-reported CHQ physical functioning (*p *= .022), with primary caregiver-reported CHQ self-esteem (*p *= .026), and with teen-reported CHQ physical functioning (*p *= .014). PIP difficulty also served as a mediator between teen PPQ pain frequency with teen-reported CHQ physical functioning (*p *= .036) and there was a trend for PIP difficulty to mediate the relationship between teen PPQ pain frequency and caregiver-reported physical functioning (*p *= .057).

#### BASC internalizing symptoms as mediator

Results for BASC internalizing symptoms as mediator were less strong than for PIP difficulty as mediator. There was a trend to significance for BASC parent depression as a mediator between primary caregiver-reported PPQ pain frequency with caregiver-reported CHQ self-esteem (*p *= .080), primary caregiver-reported PPQ pain frequency with teen-reported CHQ physical functioning (*p *= .052), and primary caregiver-reported PPQ pain frequency with teen-reported CHQ self-esteem (*p *= .109). There was a trend to significance for BASC primary caregiver rated depression as a mediator between teen-reported PPQ pain frequency with caregiver-reported CHQ self-esteem (*p *= .093) and for the relationship between teen-reported PPQ pain frequency with teen-reported CHQ physical functioning (*p *= .076). There was also a trend for BASC teen-reported anxiety to serve as a mediator between teen-reported PPQ pain frequency and teen-reported CHQ physical functioning (*p *= .086).

## Discussion

Increased understanding of the association of pain with health-related quality of life (HRQOL) is necessary for improved management of pain and other health outcomes among youth with sickle cell disease (SCD) [7] for whom HRQOL is often compromised [2]. Though pain has been linked with physical, and to a lesser extent, psychosocial domains of HRQOL for youth with SCD, we expected delineation of the role of concomitant pain variables, such as internalizing symptoms and family functioning, to better outline the relationship between pain, its associated factors, and HRQOL in this sample of adolescents with SCD. The results of this study further establish the association of sickle cell pain with physical domain and psychosocial domain of HRQOL for teens with SCD. Importantly, with variations by variable and reporter, mediation was primarily supported, particularly for disease-related parenting stress. Findings highlight a complex association of pain with HRQOL and the existence of potentially modifiable concomitant pain variables to improve HRQOL.

Because chronic and acute pain are the defining characteristics of SCD, pain serves as the cornerstone in explaining HRQOL in child, adolescent, and adult samples [7]. Our findings provide partial support for this focus on pain in studies of HRQOL in SCD. Pain frequency was strongly and consistently associated with physical aspects of HRQOL regardless of reporter (i.e. primary caregiver or teen) of pain or HRQOL. Findings were less consistent and the magnitude of the correlations was smaller for the association of pain frequency with psychosocial aspects of HRQOL as measured by the self-esteem scale of the Child Health Questionnaire (CHQ) (range of -.12 to -.34 for self-esteem compared with -.48 to -.71 for physical functioning). On the surface, the distinction between physical and psychosocial domains of HRQOL in relation to pain is not surprising as measures of physical domain of HRQOL more directly assess physiological aspects and functional impairments associated with pain. For example, the physical functioning scale of the CHQ includes responses to the question, "has it been difficult for you to do the following activities due to health problem?" Activities range from those requiring "a lot of energy" such as soccer or running to getting in and out of bed. In contrast, the self-esteem scale reflects psychosocial difficulties such as how good or bad teens felt about self, friendships, and school work. Thus, while pain should remain an important indicator of potential decrements in physical functioning, other variables including the concomitant pain variables measured in this study may better predict psychosocial aspects of HRQOL.

Noteworthy are findings of mediation for both physical and psychosocial domains of HRQOL. Whereas prior studies support the importance of psychological factors in chronic and sickle cell-related pain [14,15,33,34], this is one of the first studies in which a mediating role for both internalizing symptoms and family functioning is indicated. An interesting pattern emerged in that analyses better supported mediation models involving physical functioning (in part due to the more consistent association of physical functioning with pain frequency) and models using disease-related parenting stress as mediator. It should be considered that variation in primary caregiver and teen ratings of HRQOL (teens endorsed better physical functioning) may have played a role in the inconsistent findings. Use of multiple informants of HRQOL complicates interpretation but contributes to delineation of the multiple perspectives that may influence pain and its consequences for HRQOL [35].

The parenting stress measure used in this study, the Pediatric Inventory for Parents, was designed specifically to assess the occurrence of primarily disease-related events and the difficulty of those events for caregivers [26]. Many of the items reflect disease management activities that may be sensitive to episodes of pain in contrast to severity of pain. Thus, as these results suggest, caregiver ability to manage disease complications and treatment is integral to adolescent adaptation to SCD in the context of pain. The role of the family in adaptive outcomes for youth with SCD has been highlighted in the literature [36,37]. Our group recently reported the prospective association of parenting stress and family functioning with health outcomes [38], pointing out that families of children with SCD experience a number of socio-demographic stressors that can interact with and amplify disease-related stressors. Given the robustness of the findings, this study, therefore, further documents the importance of family functioning for physical as well as psychological adaptation.

Caregiver and teen report of pain and HRQOL and the examination of teen and family concomitant factors represent research design improvements that better inform this effort to explore HRQOL in pediatric SCD. These multiple perspectives are rarely accounted for in the literature on pediatric SCD. Variation between caregiver and teen reports of pain and HRQOL were expected based on the sickle cell and general pediatric literature [4,21,22,39], but differences in associations among variables based on reporter point to the continued importance of incorporating the family during this time of transition for teens. Caution is warranted, however, given several limitations including the small sample size of adolescent patients drawn from a comprehensive sickle cell center, reliance on retrospective reports of pain, and possible lack of utility of the CHQ in measuring self-reported HRQOL with this population. In particular, future research should incorporate prospective measurement of pain and examination of concurrent and predictive associations of pain with HRQOL to improve our understanding of mediating and potentially reciprocal associations among these variables. Furthermore, based on self-report, there were relatively fewer identified effects of pain on psychosocial aspects of functioning (and adolescent scores indicated better functioning than caregivers' reported), suggesting that use of HRQOL measures that are less susceptible to possible positive bias in self-report are required. These limitations not withstanding, the stable pattern of mediation associations indicate the importance of further consideration of internalizing symptoms and particularly parenting stress in the assessment of HRQOL and in efforts to improve functioning of youth with SCD.

## Conclusion

Studies consistently document impairments in HRQOL for youth with SCD [2], and these impairments are associated with personal and healthcare costs in pediatric populations [40]. While prospective examination of pain, concomitant variables, and HRQOL is necessary to better delineate the associations identified in this study, the findings suggest that, in addition to addressing pain management, efforts to improve HRQOL of adolescents with SCD should incorporate a focus on adolescent psychological functioning (namely reduction of anxiety and depression) and disease-related parenting stress. Particular consideration should be given to the implementation of empirically-supported interventions that improve psychological functioning of the teen by targeting attitudes about and coping with SCD and its complications [41]. Moreover, our results underscore the need to develop family focused interventions to support communication around and management of sickle cell disease complications, in particular pain, to minimize caregiver's distress in response to SCD-related events. Studies of culturally relevant disease management interventions with adolescents with SCD and family members are emerging [42], with initial results indicating the utility of a family focused model to improve disease outcomes among youth with SCD.

## Declaration of Competing interests

The authors declare that they have no competing interests.

## Authors' contributions

LPB contributed to the conception and design of the study, acquisition of data, analysis and interpretation of data, and drafting and revision of the manuscript. She has given final approval of this version for publication, CAP contributed to the conception and design of the study, acquisition of data, analysis and interpretation of data, and revision of the manuscript. He has given final approval of this version for publication, LCD participated in the analysis and interpretation of data as well as the drafting and revision of the manuscript. She has given final approval of this version for publication, CD contributed to the conception and design of the study, acquisition of data, and revision of the manuscript. He has given final approval of this version for publication.

## Consent

Written informed consent/permission was obtained from caregivers and assent was obtained from patients under 18 years of age for participation in this study and publication of research reports based on the collected data.
